# Nano-Cellulose/MOF Derived Carbon Doped CuO/Fe_3_O_4_ Nanocomposite as High Efficient Catalyst for Organic Pollutant Remedy

**DOI:** 10.3390/nano9020277

**Published:** 2019-02-16

**Authors:** Hailong Lu, Lili Zhang, Jinxia Ma, Nur Alam, Xiaofan Zhou, Yonghao Ni

**Affiliations:** 1 National-Provincial Joint Engineering Research Center of Electromechanical Product Packaging, Jiangsu Co-Innovation Center of Efficient Processing and Utilization of Forest Resources, Nanjing Forestry University, Nanjing 210037, China; 15298393737@163.com (H.L.); luo913302@163.com (L.Z.); jxma@njfu.edu.cn (J.M.); 2 Department of Chemical Engineering, University of New Brunswick, Fredericton, NB E3B 5A3, Canada; malam6@unb.ca

**Keywords:** nano-cellulose, MOF, carbon-doped CuO/Fe_3_O_4_ nanocatalyst, catalytic reduction, pollutant remedy

## Abstract

Metal–organic framework (MOF)-based derivatives are attracting increased interest in various research fields. In this study, nano-cellulose MOF-derived carbon-doped CuO/Fe_3_O_4_ nanocomposites were successfully synthesized via direct calcination of magnetic Cu-BTC MOF (HKUST-1)/Fe_3_O_4_/cellulose microfibril (CMF) composites in air. The morphology, structure, and porous properties of carbon-doped CuO/Fe_3_O_4_ nanocomposites were characterized using SEM, TEM, powder X-ray diffraction (PXRD), X-ray photoelectron spectroscopy (XPS), and vibrating sample magnetometry (VSM). The results show that the as-prepared nanocomposite catalyst is composed of Fe_3_O_4_, CuO, and carbon. Compared to the CuO/Fe_3_O_4_ catalyst from HKUST-1/Fe_3_O_4_ composite and CuO from HKUST-1, this carbon-doped CuO/Fe_3_O_4_ nanocomposite catalyst shows better catalytic efficiency in reduction reactions of 4-nitrophenol (4-NP), methylene blue (MB), and methyl orange (MO) in the presence of NaBH_4_. The enhanced catalytic performance of carbon-doped CuO/Fe_3_O_4_ is attributed to effects of carbon preventing the aggregation of CuO/Fe_3_O_4_ and providing high surface-to-volume ratio and chemical stability. Moreover, this nanocomposite catalyst is readily recoverable using an external magnet due to its superparamagnetic behavior. The recyclability/reuse of carbon-doped CuO/Fe_3_O_4_ was also investigated.

## 1. Introduction

Recently, metal nanoparticles (NPs) were widely used in the fields of biomedicine and chemical reactions due to their high selectivity and catalytic efficiency [1,2,3]. Noble-metal nanoparticles (gold, silver, etc.) [4,5,6] and non-noble-metal nanoparticles (copper, zinc, and their oxides, sulfides, etc.) [7,8,9,10] are particularly noticeable. For example, Jiang et al. [11] reported that CuO and Au domains could greatly improve the photocatalytic activity and stability of Cu_2_O cubes in the photocatalytic degradation of methyl orange (MO). Rodríguez et al. [12] reported that potassium poly(heptazine imide) (PHIK)/Ti-based metal–organic framework (MIL-125-NH_2_) composites had superior photocatalytic activity in rhodamine B (RhB) degradation under blue-light irradiation. Among the applications, metal nanoparticles can also be used for treating wastewater and drinking water due to their large surface areas and high activity [13,14]. With growing focus on the development of cost-effective, efficient catalysts, more attention is being paid to non-noble-metal catalysts, such as metal-oxide catalysts.

Metal NPs with nanometer-scale dimensions are unstable and tend to aggregate due to their high surface energy and surface area, which can lead to the loss of catalytic efficiency [15,16,17]. The concept of immobilization/stabilization of metal NPs onto support/substrates is one of the effective methods to overcome aggregation of NPs [18,19]. There are many types of substrates/matrices that can be used to support metal NPs, such as carbon [20,21], silica [22], metal oxide [23], polymers, etc. [24,25,26]. Carbon, as a support for metal nanoparticles, provides multiple accessible channels for diffusion/transport to take advantage of the excellent catalytic functionalities of metal nanoparticles [27].

In past decades, metal–organic frameworks (MOFs) attracted much attention due to their porous structures and potential applications in gas storage, molecule separation, chemical sensing, drug delivery, and catalysis [28,29,30]. Recently, MOF-based derivative catalysts received more attention because MOF-derived materials have advantages in terms of tailorable morphologies, hierarchical porosity, and easy functionalization with other heteroatoms and metal oxides [31,32]. For instance, Ji et al. used ZIF-67 as a precursor to synthesize a Pt@Co_3_O_4_ composite to improve the catalytic activity of CO oxidation [33]. Yang et al. reported that ZnO nanoparticles prepared via calcination of MOF-5 in air at 600 °C showed excellent photocatalytic degradation of rhodamine B [34]. Niu et al. synthesized a hybrid catalyst consisting of Cu/Cu_2_O NPs supported on porous carbon for the catalytic reduction of 4-nitrophenol (4-NP) using HKUST-1 as a precursor [27].

The direct pyrolysis/thermolysis treatment of MOFs is a simple and controllable method to prepare various metal oxides in a one-step process. By following it, we can successfully synthesize carbon-doped CuO/Fe_3_O_4_ composite catalysts for organic pollutant reduction. Herein, we prepared carbon-doped CuO/Fe_3_O_4_ composite catalysts via direct calcination of HKUST-1/Fe_3_O_4_/CMF composites under air. We then applied the as-prepared carbon-doped CuO/Fe_3_O_4_ composite catalysts for the catalytic reduction of 4-NP. Its catalytic performance, in comparison with a CuO/Fe_3_O_4_ composite from HKUST-1/Fe_3_O_4_ composite and CuO, is remarkably better, which is attributed to the fact that carbon doping can (1) minimize the aggregation of CuO/Fe_3_O_4_, (2) provide high surface-to-volume ratio and chemical stability for the catalyst in contact with the target pollutants, and (3) enhance the catalytic activity of the CuO/Fe_3_O_4_ catalyst. Furthermore, the carbon-doped CuO/Fe_3_O_4_ composite catalyst has excellent efficiency in the reduction of methylene blue (MB) and methyl orange (MO). The features of the carbon-doped CuO/Fe_3_O_4_ composite catalyst are as follows: CuO acts as the effective catalyst, with the doped carbon having the three functions discussed, and the magnetic Fe_3_O_4_ supports easy reuse/recycling of the catalyst using a magnet.

## 2. Experimental

### 2.1. Materials

Cellulose microfibrils (CMF) (~4.0 wt.%) from Cellulose Lab; trimesic acid (H_3_BTC), copper (II) acetate monohydrate, ethanol, and hydrochloric acid (HCl, 37%, *w*/*w*) from Sigma-Aldrich; methylene blue, ferric nitrate nonahydrate (Fe(NO_3_)_3_·9H_2_O), ferrous sulfate (FeSO_4_·7H_2_O), citric acid anhydrous, and sodium hydroxide (NaOH, 50%, *w*/*w*) from Fisher Scientific were used in this study. All other chemicals were of analytical grade and used without further purification.

### 2.2. Synthesis of Magnetic Carbon-Doped CuO/Fe_3_O_4_ Nanocomposite Catalyst

Fe_3_O_4_/cellulose microfibril (CMF) composites were prepared using chemical co-precipitation of aqueous ferrous and ferric ions, and the procedures were similar to those described in the literature [35]. CMF (7.5 g, ~4%, *w*/*w*) was diluted to 0.3 wt.% with distilled water and stirred for 5 min at 1000 rpm. The diluted CMF suspension was further treated in a sonicator (QSON-ICA) for 5 min to disperse the individual nanocellulose, followed by heating at 65 °C and bubbling with nitrogen for 10 min under magnetic stirring (500 rpm) to remove the dissolved oxygen from the CMF suspension. Then, citric acid (1 mg) and diluted HCl solution (1 mL, 2 mol/L) were added to the above solution, followed by the successive additions of 1.3 mmol Fe(NO_3_)_3_·9H_2_O and 0.65 mmol FeSO_4_·7H_2_O solid samples with nitrogen bubbling and magnetic stirring. The function of citric acid was to protect the as-prepared Fe_3_O_4_ NPs from being oxidized by the dissolving oxygen in water. A dilute NaOH solution (10 mL, 2 mol/L) was added drop-wise into the above system, followed by bubbling with nitrogen and magnetic stirring (500 rpm) for 120 min at room temperature. The obtained Fe_3_O_4_/CMF composites (using a magnet) were washed three times with distilled water in a centrifugation step (4000 rpm, 10 min).

Copper (II) acetate monohydrate was dissolved in distilled water. Fe_3_O_4_/CMF nanocomposites and H_3_BTC were dispersed in ethanol and then treated in a sonicator (QSON-ICA) for 5 min. The ethanol solution containing Fe_3_O_4_/CMF composite and H_3_BTC was added to the aqueous system. The color of the solution changed from light blue to blue-black immediately, and the system was stirred continuously for 4 h. The final HKUST-1/Fe_3_O_4_/CMF composite was washed in a centrifugation step (4000 rpm, 10 min) with distilled water and absolute ethanol three times. Then, the drying was done at 50 °C under vacuum.

The synthesized HKUST-1/Fe_3_O_4_/CMF composites were placed in a ceramic boat, then transferred to a muffle roaster, which was operated under the conditions of 500 °C, air atmosphere, and a heating rate of 20 °C/min, before being held at 500 °C for 5 h; the procedures were similar to those described in the literature [36]. After cooling to room temperature, the sample was washed several times with distilled water and absolute ethanol in a centrifugation step (4000 rpm, 10 min), and finally dried in a vacuum at 60 °C for 5 h.

### 2.3. Characterization

Transmission electron microscope (TEM) analyses were conducted with a JEOL JEM 2011 transmission electron microscope at 200 kV. Scanning electron microscope (SEM) analyses were conducted with a JEOL JSM 6400 scanning electron microscope. The powder X-ray diffraction (PXRD) patterns of the prepared samples were collected using an X-ray diffractometer with Cu-Kα radiation (40 kV, 30 mA). The patterns were recorded in the region of 2θ from 10° to 80° with a scan step of 0.02°. The chemical binding energies of the respective ions in the samples were measured using X-ray photoelectron spectroscopy (XPS, ESCALa-b220i-XL electron spectrometer, Thermo Fisher Scientific K-Alpha, UK) under 300-W Al-Kα radiation. The magnetic properties were measured with a vibrating sample magnetometer (VSM), a physical property measurement system, at 300 K, as a function of the applied magnetic field between −80 and 80 kOe. The ultraviolet–visible (UV–Vis) diffuse reflectance data were collected with a UV–Vis spectrophotometer (Evolution 201, Thermo Scientific) equipped with an integrated sphere.

### 2.4. Evaluation of Catalytic Performance

**Scheme 1:**
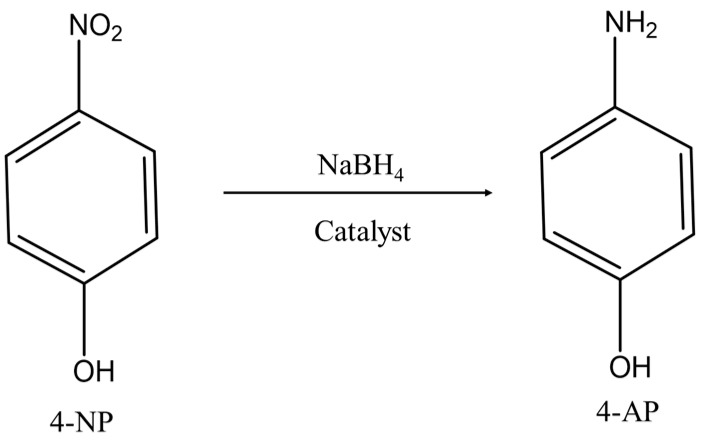
Catalytic reduction of 4-nitrophenol to 4-aminophenol.

The catalytic reduction of 4-nitrophenol (4-NP) using NaBH_4_ was chosen as the model reaction for investigating the catalytic performance of magnetic carbon-doped CuO/Fe_3_O_4_ nanocomposite catalysts. Typically, catalytic reduction of a 30-mL 4-NP solution (1 mmol/L) was carried out in a beaker (100 mL) by continuously stirring at room temperature. Upon the addition of NaBH_4_ (10 mM) into the 4-NP solution, its color changed immediately from light yellow to dark yellow due to the formation of 4-nitrophenolate ions (formed from the high alkalinity of NaBH_4_) [37]. Then, the dark-yellow color faded with time (due to the conversion of 4-NP to 4-aminophenol (4-AP)) after the addition of carbon-doped CuO/Fe_3_O_4_ nanocomposite catalyst (5 mg) (Scheme 1). UV–Vis adsorption spectra were recorded using a UV–Vis spectrophotometer in the range of 250–550 nm. When the reaction completed, the catalyst was easily separated from the solution using an external magnet due to its good magnetic performance.

During the degradation process of organic dyes, 30 mL of dye solution (10 mg/L) and NaBH_4_ (10 mM) were mixed in a beaker (100 mL) by continuously stirring at room temperature. Then, 5 mg of carbon-doped CuO/Fe_3_O_4_ nanocomposite catalyst was added to the reaction system, and the catalytic process was monitored using the UV–Vis spectrophotometer. When the reaction ended, the catalyst was readily separated from the solution using an external magnet.

## 3. Results and Discussion

### 3.1. Structure and Morphological Characterization

MOFs can be readily converted to metal-oxide composites, which take advantage of their original morphology and porosity. The as-prepared HKUST-1, HKUST-1/Fe_3_O_4_, and HKUST-1/Fe_3_O_4_/CMF composite samples were then calcinated to obtain the nanoporous metal-oxide particles, generating nanoporous CuO, CuO/Fe_3_O_4_, and carbon-doped CuO/Fe_3_O_4_ composite catalysts, respectively. Pyrolysis/thermolysis of HKUST-1/Fe_3_O_4_/CMF composites led to the formation of porous carbon-doped CuO/Fe_3_O_4_ composites.

**Figure 1:**
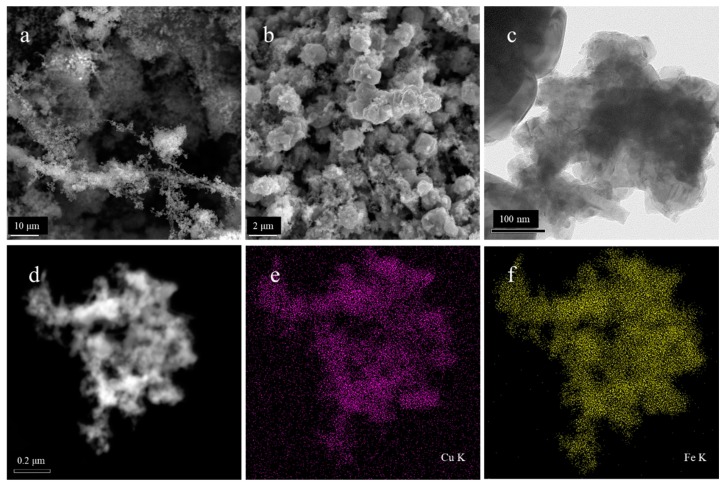
SEM images of (**a**) HKUST-1/Fe_3_O_4_/cellulose microfibril (CMF) composites and (**b**) carbon-doped CuO/Fe_3_O_4_ composite catalysts after calcination; TEM images of (**c**) carbon-doped CuO/Fe_3_O_4_ composite catalysts; (**d**–**f**) energy-dispersive X-ray spectroscopy (EDX) mapping of carbon-doped CuO/Fe_3_O_4_ composite catalysts.

The size and the morphology of the synthesized materials were investigated using a transmission electron microscope (TEM) and scanning electron microscope (SEM). Figure 1 shows the SEM and TEM images of HKUST-1/Fe_3_O_4_/CMF composites and carbon-doped CuO/Fe_3_O_4_ composites after calcination in air. HKUST-1 crystals and Fe_3_O_4_ nanoparticles were uniformly loaded onto the surface of CMF (Figure 1). The nanocellulose MOF-derived porous carbon-doped CuO/Fe_3_O_4_ composite was then obtained through a calcination process under air at 500 °C for 5 h. The Brunauer–Emmett–Teller surface area (S_BET_) of the carbon-doped CuO/Fe_3_O_4_ sample was 38.7 m^2^/g, which was much higher than that of the CuO/Fe_3_O_4_ sample (0.042 m^2^/g) obtained from the calcination of HKUST-1/Fe_3_O_4_. The resultant carbon-doped CuO/Fe_3_O_4_ composite inherited largely the original porous morphology of HKUST-1. Based on the mapping images, the conclusion was that both Cu and Fe elements dispersed well in the carbon-doped CuO/Fe_3_O_4_ composite catalyst.

**Figure 2:**
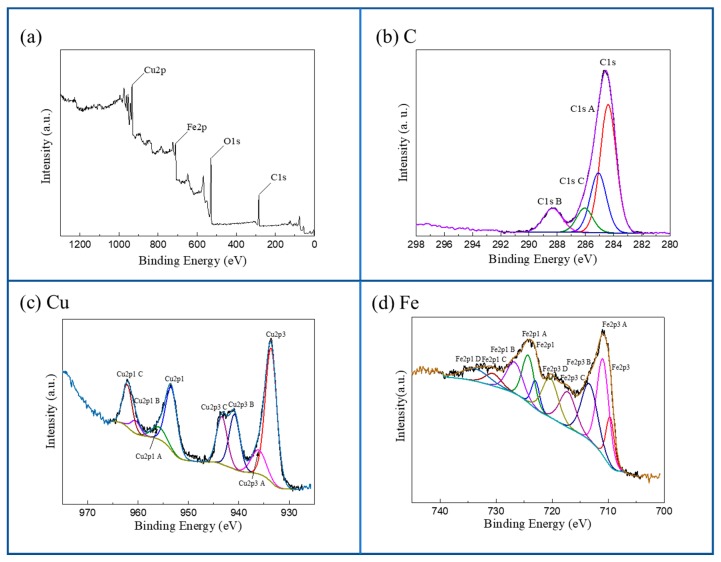
(**a**) High-resolution X-ray photoelectron spectroscopy (XPS) survey spectra of the carbon-doped CuO/Fe_3_O_4_ composite catalysts. (**b**) High-resolution XPS scans over C*1s* peaks of the carbon-doped CuO/Fe_3_O_4_ composite catalysts. (**c**) High-resolution XPS scans over Cu*2p* peaks of carbon-doped CuO/Fe_3_O_4_ composite catalysts. (**d**) High-resolution XPS scans over Fe*2p* peaks of carbon-doped CuO/Fe_3_O_4_ composite catalysts.

XPS analysis was employed to investigate the elemental composition on the surface of different composites. For the XPS spectrum of carbon-doped CuO/Fe_3_O_4_ composite catalyst (Figure 2a), the main peaks were C*1s*, O*1s*, Fe*2p*, and Cu*2p*, centered at around 285 eV, 530 eV, 720 eV, and 930 eV, respectively. The C*1s* XPS pattern of the sample is shown in Figure 2b. The spectrum contains two peaks at 285 eV and 288.5 eV, which may be attributed to carbon (*sp^2^* hybridization) in the sample, and the Cu–O–C bonds or Fe–O–C bonds, respectively. Figure 2c shows the Cu*2p* core-level XPS spectrum of the composite catalyst. The Cu*2p1* and Cu*2p3* binding energies for the composite catalyst were 952.8 and 932.7 eV, respectively, indicating the presence of CuO in the composite catalyst. Similar results were reported in the literature [38,39]. The Fe*2p3* and Fe*2p1* binding energies (Figure 2d) for the composite catalyst were 710.7 and 725.4 eV, respectively, which agrees with published results [35,40], confirming the presence of Fe_3_O_4_ in the composite catalyst.

**Figure 3:**
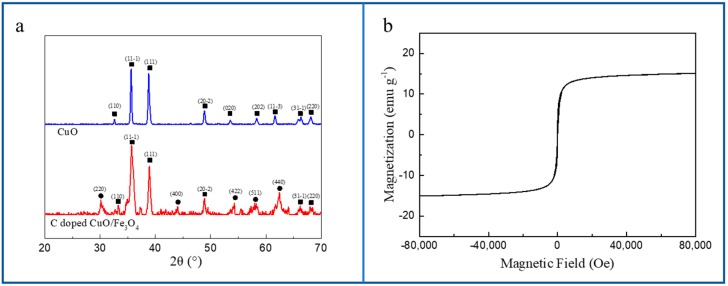
(**a**) Powder X-ray diffraction (PXRD) patterns of carbon-doped Fe_3_O_4_/CuO composite catalyst. (**b**) Magnetization curve at 300 K of carbon-doped Fe_3_O_4_/CuO composite catalyst.

The crystalline nature and the composition of the as-synthesized products were characterized using PXRD. The crystalline phases of CuO, such as (110), (11-1), (111), (20-2), (020), (202), (11-3), (31-1), and (220) are shown in Figure 3a, which are consistent with those reported in the literature [41,42]. These results support the conclusion that HKUST-1 was transformed into CuO via calcination under the present conditions. In the XRD pattern of carbon-doped CuO/Fe_3_O_4_ composite, the diffraction peaks and relative intensities were indexed to Fe_3_O_4_ (JCPD NO. 19-0629) and CuO (JCPD NO. 05-0661). This indicates that CuO and Fe_3_O_4_ were both obtained via calcining HKUST-1/Fe_3_O_4_/CMF composites under air. No other C-related impurity peaks were detected; a similar result was reported in the previous research [43].

The magnetic behavior of the carbon-doped Fe_3_O_4_/CuO composite catalyst sample was evaluated at 300 K. Its magnetization saturation value (Ms) was 15.1 emu∙g^−1^, suggesting a good magnetic property (Figure 3b). Thus, the magnetic carbon-doped Fe_3_O_4_/CuO composite catalyst could be readily separated from the reaction system using a magnet for the recycling process.

### 3.2. Catalytic Reduction

**Figure 4:**
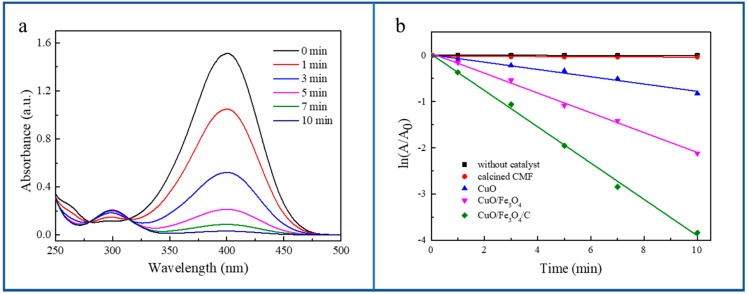
(**a**) Time-dependent ultraviolet–visible (UV–Vis) absorption spectra of 4-nitrophenol (4-NP) reduced by NaBH_4_ in the presence of carbon-doped CuO/Fe_3_O_4_ composite catalysts; (**b**) the first-order kinetic plot (absorbance at 400 nm, ln(A_t_/A_0_)) versus reaction time for the reduction of 4-nitrophenol; A_t_ and A_0_ represent the absorbance values of 4-NP at 400 nm at designated time t and t = 0, respectively.

In this study, we investigated the application of carbon-doped CuO/Fe_3_O_4_ composite catalysts for the catalytic reduction of 4-nitrophenol. In Figure 4a, the results are shown for the catalytic reduction of 4-NP to 4-AP in the presence of NaBH_4_ and carbon-doped CuO/Fe_3_O_4_ composite catalysts. The reduction process was followed based on the UV–Vis spectrophotometry. It shows that the absorbance at 400 nm (4-NP) decreased gradually as a function of time, while the absorbance at 290 nm (due to 4-AP) increased, confirming the catalytic reduction of 4-NP to 4-AP [44]. The catalytic reduction was almost complete within 10 min at room temperature, and the color of the solution changed from yellow to colorless. Similar results were also reported in the literature; Bordbar et al. [45] found that, using CuO/ZnO nanocomposites, catalytic reduction of 4-NP to 4-AP (using NaBH_4_ as the reducing agent) was completed in several minutes.

The kinetics of calcined CMF, CuO, CuO/Fe_3_O_4_, and carbon-doped CuO/Fe_3_O_4_ composites are shown in Figure 4b. In the absence of catalyst, the reduction of 4-NP by NaBH_4_ was negligible. In each case, the pseudo-first-order kinetic prevailed. The calcined CMF also had a negligible effect on the reduction of 4-NP. The rate constants (k) for CuO, CuO/Fe_3_O_4_, and carbon-doped CuO/Fe_3_O_4_ composite samples were 1.3 × 10^−3^ s^−1^, 3.6 × 10^−3^ s^−1^ and 6.5 × 10^−3^ s^−1^, respectively. In the case of CuO, the catalytic activity was the lowest due to the aggregation of CuO nanoparticles, while the catalytic efficiency of carbon-doped CuO/Fe_3_O_4_ composites was much better than that of CuO/Fe_3_O_4_, demonstrating that carbon doping is effective for enhancing the catalytic activity of the catalysts.

The catalytic reduction of 4-NP by NaBH_4_ using metal-oxide nanoparticles (CuO) has two steps [46]: (1) borohydride ions are adsorbed onto the nanoparticle surface, forming active surface-hydrogen, while 4-NP is also adsorbed onto the nanoparticle surface; (2) active hydrogen attacks the positively charged nitrogen in the nitro group of 4-NP, followed by the addition of two hydrogen atoms, producing 4-AP.

**Figure 5:**
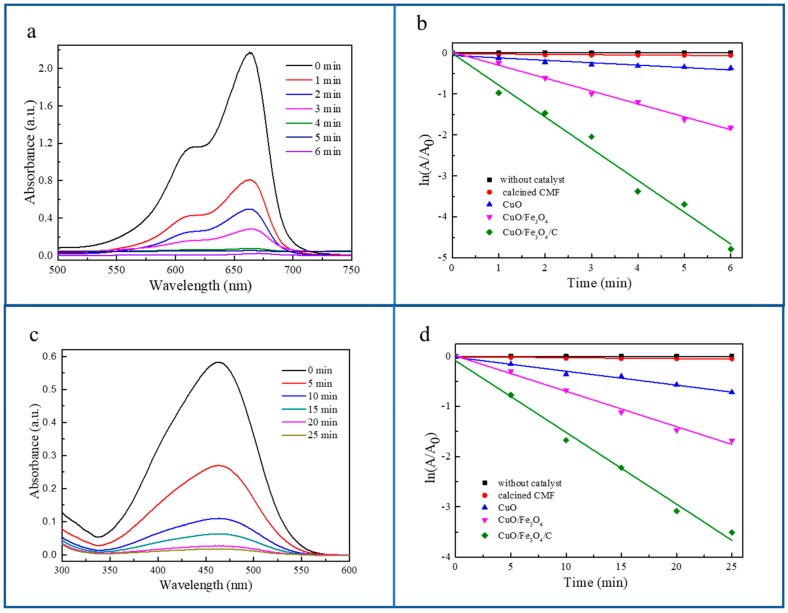
(**a**) Time-dependent UV–Vis absorption spectra of methylene blue (MB) with the carbon-doped CuO/Fe_3_O_4_ composite catalyst in the presence of NaBH_4_; (**b**) the corresponding first-order kinetic plot (absorbance at 660 nm, ln(A_t_/A_0_)) versus reaction time for the reduction of MB; A_t_ and A_0_ represent the absorbance of MB (660 nm) at designated time t and t = 0, respectively; (**c**) time-dependent UV–Vis absorption spectra of methyl orange (MO) with the carbon-doped CuO/Fe_3_O_4_ composite catalyst in the presence of NaBH_4_; (**d**) the corresponding first-order kinetic plot (absorbance at 460 nm, ln(A_t_/A_0_)) versus reaction time for the reduction of MO; A_t_ and A_0_ represent the absorbance of MO (460 nm) at designated time t and t = 0, respectively.

Cationic and anionic organic dyes were chosen to further investigate the catalytic properties of carbon-doped CuO/Fe_3_O_4_ composite catalyst. As shown in Figure 5a, for cationic dye (methylene blue), in the presence of NaBH_4_ and carbon-doped CuO/Fe_3_O_4_ composite catalyst, the absorbance at 660 nm (MB) gradually decreased as a function of time; furthermore, the catalytic reduction was completed within 6 min at room temperature (the color of the solution was colorless). 

The results from methyl orange (an anionic dye) are shown in Figure 5c. Under otherwise the same conditions, the color change (from orange to colorless) was slower than that for MB (Figure 5a, from blue to colorless). The pseudo-first-order rate law was also valid here (Figure 5d). For the carbon-doped CuO/Fe_3_O_4_ composite catalyst, the rate constant (k) was 2.4 × 10^−3^ s^−1^ for MO, while it was 12.9 × 10^−3^ s^−1^ for MB.

We compared the catalytic performance of the carbon-doped CuO/Fe_3_O_4_ composite catalyst for the reduction of 4-NP, MB, and MO, with other related ones from the literature (Table 1). As shown, the carbon-doped CuO/Fe_3_O_4_ composite catalyst showed much improved results, and the pseudo-first-order rate constant (k) for the nanocomposite catalyst from the present study was indeed consistently higher than that reported in the literature. The improved results may be attributed to the unique original morphologies associated with HKUST-1.

**Figure 6:**
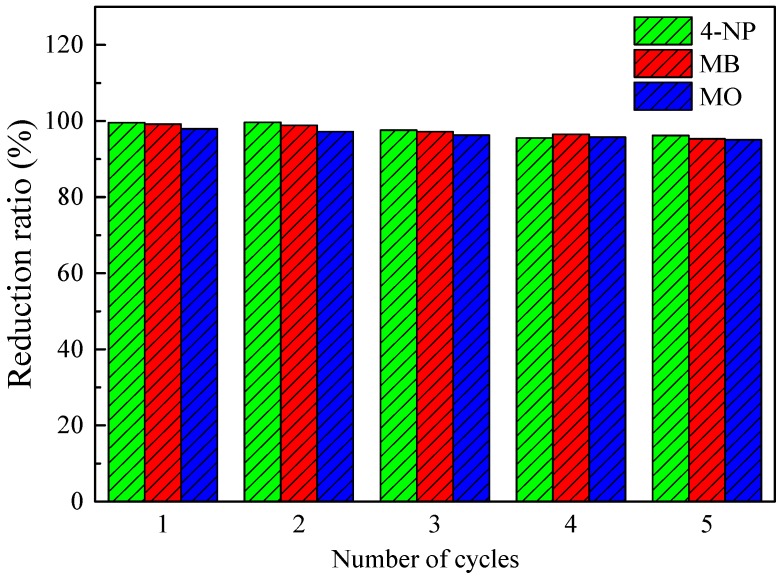
Reduction conversion ratio of 4-NP, MB, and MO after five successive cycles using the carbon-doped CuO/Fe_3_O_4_ catalyst.

From the viewpoint of practical application, the recycling/reuse of the catalyst is of critical importance. In the present study, after the catalytic degradation experiments, the magnetic carbon-doped CuO/Fe_3_O_4_ composite catalyst was readily separated from the reaction system using an external magnet. The used catalysts were collected, and rinsed with distilled water several times. After a thorough washing process, the recovered magnetic catalyst was reused in the subsequent run of catalytic reduction of 4-NP under identical conditions, and the same process was repeated five times. The results are shown in Figure 6. The catalytic performance of the magnetic carbon-doped CuO/Fe_3_O_4_ composite catalyst decreased only slightly (the 4-NP reduction ratio decreased from 100% to 96%) after five cycles. Similar results were obtained in the reuse/recycling experiments of the as-prepared magnetic carbon-doped CuO/Fe_3_O_4_ composite catalyst during the catalytic reduction of MB and MO. Therefore, the as-prepared magnetic carbon-doped CuO/Fe_3_O_4_ composite catalyst is a promising system for practical applications.

## 4. Conclusions

In this study, a nano-cellulose/MOF-derived carbon-doped CuO/Fe_3_O_4_ composite catalyst was successfully fabricated through pyrolysis/thermolysis of the HKUST-1/Fe_3_O_4_/CMF composite. The resultant carbon-doped CuO/Fe_3_O_4_ composite catalyst took advantage of the original porous morphology of HKUST-1; consequently, the carbon-doped CuO/Fe_3_O_4_ composite catalyst exhibited high catalytic activity for the reduction of 4-NP and organic dyes (MB and MO). In addition, the carbon-doped CuO/Fe_3_O_4_ composite catalyst showed good reusability/recyclability after five cycles. Notably, this strategy can be extended to the preparation of other functional MOF-based derivatives.

## Figures and Tables

**Table 1:** Comparison of the catalytic performance for the reduction of 4-nitrophenol, methylene blue (MB), and methyl orange (MO) using the carbon-doped CuO/Fe_3_O_4_ composite catalyst and other reported catalysts in the literature. NP—nanoparticle.

Pollutant	Samples	Concentration of Pollutant	Time (min)	k (s^−1^)	Reference
4-Nitrophenol	Agnp-PSAC	0.02 mM	25	3.9 × 10^−3^	[47]
	CF-AuNPs	1 mM	11	0.17 × 10^−3^	[48]
	CuO	0.1 mM	32	5.8 × 10^−3^	[49]
	CuO/Fe_3_O_4_/C	1 mM	10	6.5 × 10^−3^	This work
Methylene Blue	Au/Fe_3_O_4_@C	0.01 mM	10	5.5 × 10^−3^	[50]
	AuNPs	1 mM	9	4.0 × 10^−3^	[51]
	Fe_3_O_4_@polydopamine-Ag	0.1 mM	-	7.2 × 10^−3^	[52]
	CuO/Fe_3_O_4_/C	0.03 mM	6	12.9 × 10^−3^	This work
Methyl Orange	Ag–γ-Fe_2_O_3_	0.3 mM	30	1.53 × 10^−3^	[53]
	Ag-CuO	0.006 mM	120	0.3 × 10^−3^	[54]
	Ag@Fe	0.1 mM	14	1.6 × 10^−3^	[55]
	CuO/Fe_3_O_4_/C	0.03 mM	25	2.4 × 10^−3^	This work
